# Associations between pre-surgical daily opioid use and short-term outcomes following knee or hip arthroplasty: a prospective, exploratory cohort study

**DOI:** 10.1186/s12891-020-03413-z

**Published:** 2020-06-22

**Authors:** Justine M. Naylor, Natalie Pavlovic, Melissa Farrugia, Shaniya Ogul, Danella Hackett, Anthony Wan, Sam Adie, Bernadette Brady, Leeanne Gray, Rachael Wright, Michelle Nazar, Wei Xuan

**Affiliations:** 1 SWS Clinical School, UNSW, Locked Bag 7103, Liverpool BC, Liverpool, NSW 1871 Australia; 2 South West Sydney Local Health District, Locked Bag 7103, Liverpool BC, Liverpool, NSW 1871 Australia; 3 St George and Sutherland Clinical School, St George Hospital, Short St, Kogarah, Kogarah, NSW 2217 Australia; 4 Ingham Institute Applied Medical Research, 2 Campbell St, Liverpool, Liverpool, NSW 2170 Australia

**Keywords:** Arthroplasty, Arthroplasty, knee, Arthroplasty, hip, Opioids, Analgesia, Rehabilitation

## Abstract

**Background:**

Retrospective studies have found that daily opioid use pre-arthroplasty predicts worse longer-term service, clinical and patient-reported outcomes. Prospective studies are needed to confirm these observations. This prospective, exploratory study aimed to determine: the proportion of total knee or hip arthroplasty (TKA, THA) patients who use opioids regularly (daily) pre-surgery; if opioid use pre-surgery is associated with acute and sub-acute outcomes to 12-weeks post-surgery.

**Methods:**

Consecutive patients undergoing primary TKA or THA were prospectively enrolled pre-surgery and followed-up by telephone to 12-weeks post-surgery. Acute-care (oral morphine equivalent dosage (OMED), length of stay, discharge to inpatient rehabilitation, complications) and 12-week outcomes (Oxford Knee or Hip Score, Euroqol ‘today’ health score, current use of opioids, and complications including readmissions) were monitored. Unadjusted and adjusted Odds Ratios (ORs) (95% Confidence Interval, CI), Rate Ratios and β coefficients (standard error) were calculated.

**Results:**

Five Hundred Twenty-One patients were included (TKA *n* = 381). 15.7% (95%CI 12.6 to 18.9) used opioids regularly pre-surgery. 86.8% (452/521) were available for follow-up at 12-weeks. In unadjusted analyses, pre-surgical opioid use was significantly associated with higher average acute daily OMED [β 0.40 (0.07), *p* <  0.001], presence of an acute complication [OR 1.75 (1.02 to 3.00)], and ongoing use of opioids at 12-weeks [OR 5.06 (2.86 to 8.93)]. After adjusting for covariates, opioid use pre-surgery remained significantly associated with average acute daily OMED [β 0.40 (0.07), *p* <  0.001] and ongoing use at 12-weeks [OR 5.38 (2.89 to 9.99)].

**Conclusion:**

People who take daily opioids pre-surgery have significantly greater odds for greater opioid consumption acutely and ongoing use post-surgery. Adequately powered prospective studies are required to confirm whether pre-surgical opioid use is or is not associated with poorer joint and quality of life scores or a complication in the short-term.

## Background

Australia [1, 2], along with other countries [3, 4], is currently considered to have a major pharmaceutical opioid problem. Hospitalisation due to opioids [5] and mortality due to overdose [6] have increased since 2000–01. Whilst the Royal Australian College of General Practitioners consider opioids an important part of the armoury for managing pain, their use is cautioned for their association with dose-dependent harm including dependence, withdrawal, falls, and cognitive effects, and rather should be administered as a last resort [7]. It is well-recognised that people suffering from chronic knee or hip arthritis are prescribed strong opioid therapy [8–10], and this is despite i) an absence of strong evidence of clinically relevant benefit over active or inactive control therapies [11, 12], and ii) concern over their safety [13]. Moreover, many people – up to 24% as estimated by a recent systematic review [14] - with end-stage arthritis and awaiting total knee or hip arthroplasty (TKA, THA) are taking opioids prior to surgery and this appears to have implications for recovery. Retrospective studies indicate pre-surgical opioid use is both a strong risk factor for persistent use post-surgery [15–17] and is associated with higher risk of readmission, revision surgery and worse outcomes generally including periprosthetic joint infection [18–23]. The aforementioned systematic review concluded that pre-surgical opioid use (versus no use) has a moderate effect on absolute index joint scores (pain, function or both) 6–58 months post-surgery though relative improvements are similar [14]. Very little is known about index joint scores and even health-related quality of life scores within 6-months of surgery. Understanding early recovery is important given that long-term recovery may be confounded by other factors, especially if people continue to take opioids long-term after surgery.

Given the above concerns, opioid tapering is recommended for chronic opioid users prior to elective surgery [18, 24], thus, tapering may be a useful strategy for chronic opioid users prior to arthroplasty. However, prior to recommending what is likely to be a resource-intensive, multi-pronged approach [24, 25], evidence from prospective trials, controlling for important confounders, are needed to accurately depict both the extent of the problem and the associated harms.

The aims of this prospective, exploratory study were multiple. Using a single-centre, Australian public hospital cohort, we aimed to:

i) determine the proportion (plus 95% confidence interval (CI)) of regular (daily) opioid users at the time of surgery in a population undergoing primary TKA or THA
ii) determine whether regular opioid use prior to surgery is associated with worse acute-care (higher opioid consumption, complications, longer hospital length of stay, admission to inpatient rehabilitation) and sub-acute (12-week) outcomes (complications including any readmissions, ongoing use of opioids, and worse patient-reported index joint scores and health-related quality of life).

## Methods

### Design and ethical approval

This study was a planned, secondary analysis of a quasi-experimental (controlled before (‘historical’) vs after (‘intervention’) design) quality improvement (QI) initiative. The QI initiative was developed by a multidisciplinary steering committee aimed at introducing earlier ambulation (commencing Day 0, intervention cohort) following TKA or THA. The study was approved by a Lead Human Research Ethics Committee. All patients were provided with a Patient Information Sheet (in various languages) prior to their pre-operative assessment. Whilst all patients were included in the acute-care QI initiative, only patients who provided informed, verbal consent (pre-operatively and at the time of follow-up), as was approved by the ethics committee, were included in the follow-up. A record of who provided verbal consent and who did not was kept in an excel file by the chief investigator.

### Setting

High-volume (> 600 TKA or THA procedures annually), public arthroplasty centre located in a region with vast cultural and linguistic diversity (CALD).

### Patient screening and data collection

Consecutive patients were screened for eligibility by an investigator at the pre-admission visit typically 2–6 weeks prior to surgery. Interpreters were used when indicated. All patients undergoing primary unilateral or bilateral TKA or THA were considered eligible, regardless of their level of proficiency in English. As was routine for the centre, patients completed patient reported outcome measures (PROMs) - Oxford Knee or Hip Scores (OKS, OHS) [26], and the Euroqol 5 Dimension (EQ-5D) health related quality of life survey [27], of which only the today health visual analogue scale (0–100 cm) (EQVAS) was used. The PROMS data were collected using iPads or via email links and compiled centrally as part of the Australian National Joint Replacement Registry PROMS project [https://www.monash.edu/__data/assets/pdf_file/0019/1571113/Grace-ODonohue20181109_AOANJRRPROMs_Monash.pdf]. Using a data extraction pro forma, trained research officers (ROs) extracted patient information from the paper-based and electronic medical record, including: joint (knee or hip); age; sex; comorbidities (diabetes, chronic respiratory disease, heart disease, hypertension, any central nervous system condition, any diagnosed mental health condition; any back pain or other lower limb problems); body mass index (BMI); American Society of Anesthesiologists (ASA) score; unilateral or bilateral surgery; primary diagnosis (osteoarthritis or other); education level (Years 8 or less; Years 9–10; Years 11–12; degree qualified); interpreter required; smoking status (current or past/never); and consumption of more than two standard drinks of alcohol per day. Regular (daily) opioid use was determined by patient-reported opioid use at initial assessment upon entry to the waitlist, updated where possible at subsequent waitlist review assessments as well as at the time of review by the anaesthetist in the preadmission clinic, and included any prescription-based opioid medication for any indication.

After the patient had undergone surgery, ROs also extracted acute-care data including: discharge destination (home/usual/relative residence vs inpatient rehabilitation); any complication requiring ongoing management or monitoring [major joint (deep surgical site infection (SSI), wound bleed/haemarthrosis, dehiscence, nerve injury, dislocation, intraoperative fracture); minor joint (persistent wound ooze, suspected SSI, blistering); major non-joint (death, myocardial infarction, symptomatic venous thromboembolism (VTE), aspiration, chest infection, excessive non-joint bleeding, fall with injury, cerebrovascular accident, renal injury); minor non-joint (cellulitis, delirium, atelectasis, urinary tract infection, electrolyte disturbance, polyuria, ulcers, urinary retention)]; length of stay (LOS) (days); total and daily oral morphine equivalent dose (OMED) including intra-operative doses using a recognised algorithm for conversion [28]. We also calculated the total costs of investigations (pathology and imaging tests) incurred for each patient over the course of the acute admission. [Refer Additional file 1 for associated methodology.]

Sub-acute outcomes were obtained by telephone follow-up at 4 and 12-weeks post-surgery by ROs following time-specific study pro forma, using interpreters as required. Patient-recalled readmissions were checked and corroborated by medical record review if the readmission occurred within the same Local Health District. Outcomes included: complications or readmissions (any cause) in the first 12-weeks [complications included as above plus others (major joint (manipulation under anaesthetic)); minor joint (clip/stitch irritation, stiffness without manipulation under anaesthetic); major non-joint (constipation requiring readmission, symptomatic anaemia requiring intervention); minor non-joint (new incontinence)]; the OKS or OHS and the Euroqol survey at 12-weeks, and; continued use of opioids at 12-weeks (for index joint as well as any indication). Patients not contacted after multiple attempts and within the first 16 post-operative weeks were considered lost to follow-up (LTFU).

### Sample size and analyses

The sample size was dictated by the sample required for the QI initiative (~ 500). Here, a sample of 500 was considered adequate for providing a robust estimate of the proportion (95% CI) of patients deemed regular opioid users at the time of surgery (exposure variable) as well as exploring the association between the latter and the various aforementioned outcomes whilst adjusting for potential confounders. Assuming the exposure variable applied to 25% (*n* = 125) of the cohort as per a recent systematic review [14], we planned to include up to 12 covariates in each regression model. This would achieve an observation (exposure): covariate ratio of ~ 10:1 for multiple logistic regression modelling for the binary outcomes, and much higher subject:variable ratios (~ 42:1) for multivariable linear regression modelling for continuous outcomes and Poisson regression for LOS. These ratios are considered adequate in the context of confounder adjustment, although ratios as low as 5–9 are also considered acceptable for logistic regression [29, 30].

Outcome variables and other descriptor variables were reported as mean (standard deviation (SD), median (Interquartile range (IQR)), and percentage (95%CI)) as appropriate. Between- group comparisons (opioid vs non-opioid users) of baseline characteristics and 12-week outcomes were undertaken using unpaired t-tests, χ^2^ tests or Wilcoxon-Mann-Whitney tests. Prior to undertaking definitive analyses, the continuous dependent outcomes were assessed for normality. Log transformations were undertaken for those outcomes not normally distributed or they were converted to binary outcomes if the log transformation did not achieve normality. Poisson regression was used for count outcomes. For the unadjusted (bivariate) and adjusted (multiple regression) analyses, the Odds Ratio (OR) was determined for most binary outcomes, the β coefficient (and standard error (SE)) was used for log transformed outcomes, and the Rate Ratio was used for the count outcome. Regardless of the level of significance of the unadjusted association, all dependent variables were tested in adjusted models given the exploratory nature of the study. The covariates to be included in the risk-adjustment models were based on previously published studies in this area [14, 16, 18, 23] as well as local knowledge of factors affecting opioid use or the outcomes of interest. Where it was appropriate to do so, the same covariates were used in each model. As the two-phase QI initiative (the overarching study) was unlikely to influence the relationships between regular opioid use and the stated outcomes, ‘study period’ was not included as a covariate.

As recommended for exploratory studies [31], no adjustment to the significant *p*-value was made for multiple comparisons. No imputation of missing covariate or outcome data was undertaken. The data were stored and cleaned in Microsoft® Excel® for Office 365; analysis was undertaken in SAS Version 9.4 (SAS Institute Inc. Cary, NC, USA).

## Results

**Fig. 1:**
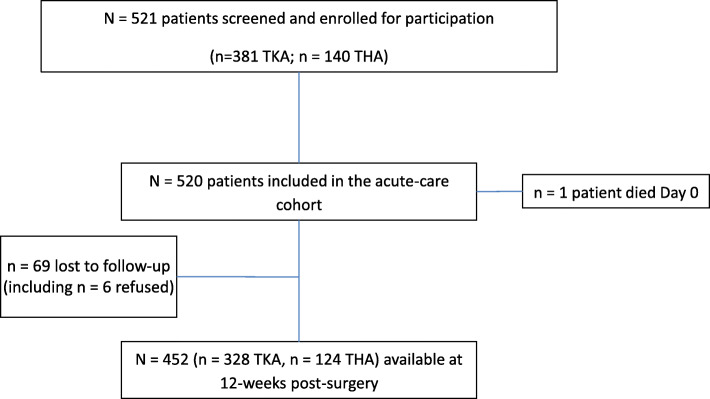
Cohort ascertainment and retention. Legend: TKA = total knee arthroplasty; THA = total hip arthroplasty; N or *n* = sample size

521 people underwent primary TKA or THA (*n* = 381 TKA; *n* = 140 THA; *n* = 503 unilateral) over the 10-month study period (23rd July 2018 to 29th May 2019). Cohort derivation and retention to 12-weeks post-surgery are summarised in Fig. 1. 96.5% (*n* = 503) and 86.8% (*n* = 452) were available for follow-up at 4- and 12-weeks post-surgery (one patient died acutely). Compared to those retained, those LTFU at 12-weeks were similar in most key characteristics: female sex [66.7 (LTFU) vs 65.3% (retained), *p* = 0.82], procedure [TKA 76.8 vs 72.6%, *p* = 0.46], pre-surgery BMI [32.3 (6.1) vs 33.0 (6.8), *p* = 0.33], pre-surgery Oxford scores [17.7 (8.8) vs 18.2 (8.2), *p* = 0.69], pre-surgery EQVAS [70 (IQR 29) vs 65 (IQR 30), *p* = 0.76], age [70.2 (10.2) vs 67.5 (9.5) yr, *p* = 0.04], and regular opioid users pre-surgically [15.9 vs 15.7%, *p* = 0.98].

Table 1 summarises the cohort according to the exposure variable. 15.7% (95% CI 12.6 to 18.9) of the cohort were regular users of opioids pre-surgery. A significantly greater proportion of the pre-surgical opioid user group were undergoing THA, had an ASA score of 3 or 4, did not require an interpreter, had a mental health condition, and were a current smoker. The pre-surgery opioid user group also had a significantly higher mean BMI and a lower mean Oxford score. Education level was also significantly different between the groups with a greater proportion in the group taking opioids pre-surgically reporting a higher level of education attainment. Opioid users also had a slightly shorter wait time for surgery.

**Table 1:** Characteristics of cohort by pre-surgical opioid use status

	Non-opioid user *N* = 439^a^	Opioid user *N* = 82^a^	*P*-value
Wait time for surgery, days	332.6 (53)	307.0 (93)	0.017
Age	68.0 (9.8)	67.3 (9.0)	0.536
Male	154 (35.1)	26 (31.7)	0.556
Body mass index	32.5 (6.3)	35.1 (8.4)	0.001
Total hip arthroplasty	108 (24.6)	32 (39.0)	0.007
Unilateral procedure	423 (96.4)	80 (97.6)	0.583
Osteoarthritis	420 (95.7)	78 (95.1)	0.824
ASA score 3 or 4 (*n* = 517)	198 (45.5)	48 (58.5)	0.030
Education level (*n* = 498)			0.028
Yr 8 or below	127 (30.3)	12 (15.2)	
Yrs 9–10	169 (40.3)	38 (48.1)	
Yrs 11–12	94 (22.4)	25 (31.7)	
Degree	29 (6.9)	4 (5.1)	
Employed (*n* = 460)	79 (20.2)	13 (18.8)	0.794
Daily simple analgesics (*n* = 520)	241 (55.0)	46 (56.1)	0.856
Daily nonsteroidal medication (*n* = 518)	100 (22.9)	25 (30.9)	0.123
Requiring an interpreter	141 (32.1)	14 (17.1)	0.006
Current smoker	31 (7.1)	16 (19.5)	0.0003
Alcohol use daily (2 or more) (*n* = 499)	18 (4.3)	6 (7.6)	0.25
Other lower limb or back pain (*n* = 499)	250 (59.5)	52 (65.8)	0.29
Heart disease	106 (24.2)	22 (26.8)	0.60
Diabetes (1 or 2)	103 (23.5)	18 (22.0)	0.766
Chronic lung disease	83 (18.9)	19 (23.2)	0.37
Mental health condition	66 (15.0)	23 (28.1)	0.004
Central nervous system disorder	41 (9.3)	7 (8.5)	0.818
Hypertension	297 (67.7)	59 (72.0)	0.443
Oxford knee or hip score^b^ pre-surgery, *n* = 422	18.7 (8.3)	15.2 (7.5)	0.001
EQ VAS^c^ pre-surgery, *n* = 421	64.4 (22.0)	60.2 (20.0)	0.146

^a^maximum for any variable; data are n (%) or mean (SD); (*n* = sample, denotes when sample incomplete); ^b^ higher scores are better (maximum 48); ^c^ Higher score is better (maximum 100)

Table 2 summarises the mean, median or proportions as appropriate for each outcome. Table 3 summarises the results for unadjusted and adjusted analyses excluding the OMED results. The results of the complete models, including specification of the covariates for each model, are provided in the Additional files 2, 3, 4, 5, 6, 7, 8, 9 (Tables 1S, 2S, 3S, 4S, 5S, 6S, 7S, 8S). Due to the lower than expected frequency of regular opioid users pre-surgery, the maximum number of covariates in the models was restricted to nine. Approximately 25% of the cohort (predominantly non-English speakers) did not complete PROMS pre-surgery, reflecting the voluntary and unsupervised nature of completion using a centralised digital system. Consequently, PROM data were not used as covariates for any outcomes.

**Table 2:** Acute and 12-week outcomes by pre-surgical opioid use status

	Non-opioid *N* = 439 max	Opioid user *N* = 82 max	*P*-value
Oral morphine equivalent dose, mean			
log	3.81	4.19	< 0.001
Daily, mg	53.2 (36)	87.2 (81.2)	< 0.001
Total, mg	227 (174)	406 (329)	< 0.001
LOS, days, mean (SD)	4.7 (2.4)	5.1 (2.8)	0.218
Referral to inpatient rehabilitation, n (%)	36 (8.2)	12 (14.8)	0.059
Acute complications, n (%)	80 (18.2)	23 (28.1)	0.040
Major joint	9 (2.1)	1 (1.2)	1.000
Minor joint	35 (8.0)	10 (12.2)	0.210
Major non-joint	11 (2.5)	4 (4.9)	0.240
Minor non-joint	35 (8.0)	13 (15.9)	0.024
Cost of investigations, $			
median (IQR)	156.55 (143)	189.80 (230)	0.003
mean (SD)	232.62 (260)	316.81 (329)	0.033
Complications or readmissions to 12 weeks, n (%)	167 (38.0)	35 (42.7)	0.428
(Readmissions)	(31 (7.1))	(9 (11.0))	
Oxford score 12 weeks, n (%) in lower quartile^a^	96 (25.7)	18 (26.1)	0.950
Oxford score^c^, mean (SD), 12 weeks^a^	37.4 (7.4)	36.1 (7.4)	0.182
EQVAS 12 weeks, n (%) in lower quartile^b^	92 (25.1)	23 (33.8)	0.130
EQVAS^d^ mean (SD) 12 weeks^b^	75.2 (17.6)	71.6 (18.8)	0.133
Ongoing opioids at 12 -weeks, any reason, n (%) (for index joint joint)	39 (9.2) (32) (82.0)	27 (33.8) (17) (63.0)	< 0.001

^a^15% of the data missing; ^b^ 17% of the data missing; $ = Australian dollars; ^c^higher scores are better (maximum 48); ^d^Higher score is better (maximum 100)

**Table 3:** Unadjusted and adjusted outcomes of regression modelling

	Unadjusted Odds Ratio or Rate Ratio (95% CI)		Adjusted Odds Ratio or Rate Ratio (95% CI)	
	Opioid user	*P*-value	Opioid user	*P*-value
Length of stay	1.09 (0.98 to 1.21)	0.120	1.05 (0.94 to 1.17)	0.365
Acute complications	1.75 (1.02 to 3.00)	0.042	1.66 (0.94 to 2.94)	0.082
Referral to inpatient rehabilitation	1.95 (0.97 to 3.93)	0.063	1.86 (0.80 to 4.28)	0.147
Complications or readmissions to 12 weeks	1.21 (0.75 to 1.96)	0.429	1.05 (0.64 to 1.73)	0.845
Oxford score 12 weeks, % in lower quartile*	1.02 (0.56 to 1.83)	0.950	0.96 (0.51 to 1.80)	0.897
EQVAS 12 weeks, % in lower quartile**	1.53 (0.88 to 2.66)	0.134	1.41 (0.77 to 2.57)	0.264
Ongoing opioid use at 12 -weeks	5.06 (2.86 to 8.93)	< 0.001	5.38 (2.89 to 9.99)	< 0.001

### Association between pre-surgical opioid use and daily acute OMED

The daily acute OMED outcome was log transformed. On unadjusted and adjusted analyses, pre-surgical opioid use was associated with significantly greater daily opioid consumption acutely [unadjusted - β 0.402 (SE 0.074), *p* <  0.001; adjusted - β 0.395 (SE 0.070), *p* <  0.001].

### Association between pre-surgical opioid use and acute complications

On unadjusted analysis, pre-surgical opioid use was associated with the presence of an acute complication [OR 1.75 (1.02 to 3.00)]. This was mainly due to a higher rate of minor non-joint complications amongst the opioid users (Table 3). The association between any complication and pre-surgical opioid use was not significant on adjusted analyses [OR 1.66 (0.94 to 2.94)]. Consistent with the higher complication rate, pre-surgery opioid users had significantly higher investigation costs [mean difference $84.19 (95%CI 6.92 to 161.46) (unadjusted)].

### Association between pre-surgical opioid use and LOS

On unadjusted and adjusted analyses, LOS was not associated with pre-surgical opioid use [unadjusted Rate Ratio 1.09 (0.98 to 1.21); adjusted Rate Ratio 1.05 (0.94 to 1.17)].

### Association between pre-surgical opioid use and referral to inpatient rehabilitation

On unadjusted analysis, referral to inpatient rehabilitation was greater amongst pre-surgical opioid users, but the association was not statistically significant [OR 1.95 (0.97 to 3.93)]. The association remained non-significant on adjusted analyses [OR 1.86 (0.80 to 4.28)].

### Association between pre-surgical opioid use and Oxford score at 12-weeks

Oxford scores were analysed as a dichotomous variable (lower quartile vs remaining quartiles). On unadjusted and adjusted analyses, the association between pre-surgical opioid use and proportion of people in the lowest quartile was not significant [unadjusted OR 1.02 (0.56 to 1.83); adjusted OR 0.96 (0.51 to 1.80)].

### Association between pre-surgical opioid use and EQVAS at 12-weeks

As for Oxford scores, the EQVAS scores were analysed as a dichotomous variable (lower quartile vs remaining quartiles). On unadjusted analysis, pre-surgical opioid users had a higher proportion in the lowest quartile of EQVAS scores, but the association was not significant [OR 1.53 (0.88 to 2.66)]. The association remained non-significant on adjusted analyses [OR 1.41 (0.77 to 2.55)].

### Association between pre-surgical opioid use and complications or readmission to 12-weeks

Despite a trend for a greater rate of complications or readmission (including acute complications to 12-weeks and any readmissions), on unadjusted and adjusted analyses, pre-surgical opioid use was not significantly associated with this outcome [unadjusted OR 1.21 (0.75 to 1.96); adjusted OR 1.05 (0.64 to 1.73)]. Most readmissions (95%) were verified by chart review.

### Association between pre-surgical opioid use and opioid use at 12-weeks

Over one-third of pre-surgical opioid users remained on opioid therapy 12-weeks post-surgery - 63% of whom (17/27) reported the opioid use was for their index joint. In contrast, 9% of non-opioid users pre-surgically reported opioid use at 12-weeks, 82% of whom (32/39) reported their use was for the index joint. On unadjusted and adjusted analyses, pre-surgical opioid use was associated with a significantly greater OR for ongoing opioid use at 12-weeks [unadjusted OR 5.06 (2.86 to 8.93); adjusted OR 5.38 (2.89 to 9.99)].

## Discussion

Observations from this exploratory, prospective study corroborate data obtained elsewhere using retrospective study designs, but also provide new, potentially contrary insights about the early post-surgical risks associated with opioid use pre-surgery. We observed, as have others, that people undergoing primary TKA or THA who are regular opioid users pre-surgery have higher odds of ongoing opioid use post-surgery [15–17]. A novel finding was the greater daily consumption of opioids acutely by regular opioid users even after accounting for other factors. Few others have captured opioid use acutely [32, 33]; confounding factors such as LOS or complications have not been accounted for so comparisons are difficult. We acknowledge (and the same uncertainty applies to previous studies [32, 33]) that it is unknown whether the greater consumption acutely is clinician-driven (i.e. is pre-emptive as the anaesthetist a priori prescribes higher doses to those perceived as opioid tolerant) or patient-driven (i.e. is reactionary, with the patient simply requiring more to alleviate pain given their possible dependency).

In contrast to previous investigations [18–23, 33], chronic opioid use pre-surgery was not strongly associated with complications after accounting for other factors. The directional changes (higher rates amongst pre-surgical opioid users) were consistent with previous research, however, so a larger sample may have secured a significant result. Differences in follow-up duration, however, may also explain the discrepancy. Longer follow-up times are themselves confounded by time so other factors (for example, persistent opioid use) may be contributing to complications seen in studies with long follow-up, and our follow-up was too short to gauge insights into prosthesis longevity, for example. The degree of opioid dependence pre-surgery may also be a factor. Recent large studies from the United States (US) using insurance claims data demonstrate that adverse events or readmissions are more common in those who used opioids for longer periods pre-surgery [19, 22]. At the very least, our data suggest that minor, non-joint complications may be driving an association between chronic pre-surgical use and acute complications. Whilst such events appear to increase the costs of care in the form of investigation costs, their clinical relevance, certainly for the long-term success of the surgery, may be less concerning than joint-specific issues. That the extra risk with pre-surgical opioid use appeared to be associated with only minor complications may explain why we observed a similar LOS across the groups whilst others have demonstrated that pre-surgery opioid use is a predictor of a longer LOS [32–34].

We did not find that pre-surgery opioid use was associated with worse patient-reported index joint scores or health-related quality of life in the early sub-acute period. That we were unable to include pre-surgery PROMs in the modelling (due to a high proportion of missing data) does not likely explain this particular lack of association as patients in the pre-surgical opioid group had worse scores prior to surgery (significantly worse for the Oxford scores) and this would likely have meant this group had a larger relative improvement in their scores at 12-weeks. It is possible, then, we would have found that pre-surgical use was associated with greater improvement. An aforementioned systematic review concluded opioid use pre-surgery does undermine joint-specific scores at 6-months or more post-surgery [14], but only in terms of absolute recovery and not relative change. A recent non-randomised, retrospective study concluded that PROMs were better 6–12 months post-surgery amongst chronic opioid users who reduced their dose prior to surgery compared to those who did not; and that the former were similar to an opioid naive group [35]. We contend then that the association between pre-surgical opioid use and PROMS post-surgery, like the association with complications, is complex and in part depends on the length of follow-up but also possibly on the level of dependence or the degree of tapering that has occurred (if any) pre-surgery. Our study only established ‘use’ pre-surgery and not ‘how much’ and for ‘how long’.

It is of interest to note that almost 34% of chronic opioid users remained opioid users at 12-weeks post-surgery and this is at a time when symptomatic and functional recovery has already undergone large and clinically relevant improvements [36–38]. It is also noteworthy that many reported the ongoing use for problems other than their index joint. A recent population-based Australian study observed that amongst a cohort of people who were prescribed opioids for non-cancer pain, 2.6% were persistent users 12-months after the initial prescription [39]. Considering these observations together, we contend that the rate we observed at 12-weeks is alarming given that many of our patients were using opioids at the time they were waitlisted for surgery almost 12-months prior. If arthroplasty recipients are at higher risk of persistent opioid use than persons suffering from other forms of non-cancer pain, arthroplasty services are arguably obliged to educate recipients and their families as well as their general practitioners about the need to monitor use and have a plan for weaning [40]. This is in addition to acute care practices designed to spare opioid analgesia [41].

Pooled data from the US estimate that approximately 24% of people undergoing TKA or THA are chronic opioid users pre-surgery [14]. Our data indicate the proportion (~ 16%) to be slightly lower. The disparity in observed rates may in part be related to how pre-surgical opioid use was defined; we note that in the aforementioned review the definition of ‘opioid use pre-surgery’ varied widely, incorporating use within 6-weeks of surgery or any use within 2-years of surgery [14]. There may be other contributing factors as well. The present study was conducted in a local health district with vast ethno cultural diversity and constitutes one of the most multicultural communities in Australia [42]. This is significant because Australian data suggest lower consumption of pharmaceutical and other drugs among CALD communities [43], consistent with international literature on minority communities [44, 45]. Our cultural and language diversity also likely explains our arguably counterintuitive observation that a greater proportion of people who were opioid users pre-surgery had a higher level of education than non-users. Approximately 30% of the cohort were non-English speakers and lack of English proficiency is often associated with low level education in our CALD population. The majority of the non-English speakers were in the non-user group, hence, we see a higher level of education amongst the pre-surgical opioid users (mostly English speakers). Another explanation for our comparatively low rate of pre-surgical opioid use is that opioid use may be region-specific. Data from the US data suggest that opioid use prior to arthroplasty varies by region from 8.9 to 26.4% [46]. An additional or alternative explanation is that because the pooled data in the systematic review were based on data entirely from populations within the US where the level of opioid prescription observed is higher generally [47], our differences may simply be reflective of this phenomenon.

Strengths of our study include the provision of novel data from an Australian perspective capturing almost 100% of those screened with little loss to follow-up. The prospective design allowed us to include relevant covariates not included in retrospectively designed studies such as need for an interpreter or complication status (for PROMs outcomes), and we included non-English speakers, improving the generalisability of our results. Limitations are several and include that we do not know whom was opioid dependent pre-surgery and we could not test for a dose-response as data on dose pre-surgery was unclear. We relied on patient-reported use of opioids both prior to surgery and after discharge. The sample was small compared to the administrative datasets used in retrospective studies and this may have affected our power to control for many covariates, especially those with low rates of occurrence such as alcohol use and bilateral surgery. That said, the incidences in both groups were similar. We were unable to include PROMS as covariates in our modelling, we examined multiple outcomes without adjusting significant probability values, and our results were limited to a single centre and the short term.

## Conclusion

This exploratory study confirms previous observations that people who take daily opioids pre-surgery have significantly greater odds for greater opioid consumption acutely and ongoing use post-surgery. Given the sample size limitations here and the many covariates that need to be considered in modelling, adequately powered prospective studies are required to confirm whether pre-surgical opioid use is or is not associated with poorer joint and quality of life scores or a complication in the short-term.

## Supplementary information

**Additional file 1.** Contains Methodology for estimating cost of investigations.

**Additional file 2: Table 1S.** Log transformed procedure for daily morphine equivalent.

**Additional file 3: Table 2S.** Multiple logistic regression for acute complications.

**Additional file 4: Table 3S.** Poisson regression for length of stay.

**Additional file 5: Table 4S.** Multiple logistic regression for discharge to inpatient rehabilitation.

**Additional file 6: Table 5S.** Multiple logistic regression for complication or readmission to 12-weeks.

**Additional file 7: Table 6S.** Multiple logistic regression – Proportion in lowest quartile of Oxford scores at 12 weeks.

**Additional file 8: Table 7S.** Multiple logistic regression – Proportion in lowest quartile of EQ VAS scores at 12- weeks.

**Additional file 9: Table 8S.** Multiple logistic regression for ongoing opioid use at 12-weeks.

## Data Availability

The datasets generated and/or analysed during the current study are available from the corresponding author on reasonable request.

## Abbreviations

ASA: American Society of Anesthesiologists
BMI: Body mass index
CALD: Cultural and linguistic diversity
CI: Confidence interval
EQ-5D: Euroqol 5 dimension
EQVAS: Euroqul visual analogue scale
IQR: Interquartile range
LOS: Length of stay
LTFU: Lost to follow-up
OHS: Oxford Hip Score
OKS: Oxford Knee Score
OMED: Oral morphine equivalent dosage
OR: Odds ratio
PROMS: Patient reported outcome measures
QI: Quality improvement
SD: Standard deviation
SE: Standard error
SSI: Surgical site infection
THA: Total hip arthroplasty
TKA: Total knee arthroplasty
