# Supplementary material for: Associations between pre-surgical daily opioid use and short-term outcomes following knee or hip arthroplasty: a prospective, exploratory cohort study
Source: BMC Musculoskelet Disord. 2020 Jun 22;21:398. doi: 10.1186/s12891-020-03413-z (PMC7310486; doi:10.1186/s12891-020-03413-z)
Supplement: Supplementary file 1 — Additional file 1. Contains Methodology for estimating cost of investigations. [file 12891_2020_3413_MOESM1_ESM.docx]

**ADDITIONAL FILE 1**

**Associations between pre-surgical daily opioid use and short-term outcomes following knee or hip arthroplasty: a prospective, exploratory cohort study**

Justine M Naylor (PhD, BAppSc (Phty)), Natalie Pavlovic (BAppSc (Phty) Hons 1), Melissa Farrugia (BAppSc (Phty)), Shaniya Ogul (BN), Danella Hackett (M Physio, BAppScEXSS), Anthony Wan (MBBS, FANZCA), Sam Adie (BSc(Med) MBBS(Hons) MSpMed MPH PhD FRACS), Bernadette Brady (PhD, MManTher, BAppSc (Phty) Hons 1), Leeanne Gray (Grad Dip Nursing management), Rachael Wright (BAppSc (OccTherapy)), Michelle Nazar (RN), Wei Xuan (MSc MAppStat PhD)

1. Methodology for estimating cost of investigations

Counts of the type and number of investigations (pathology and imaging tests) were extracted for each patient from the electronic medical record. The test data contain the unique procedure identifier, procedure name, time, and unique patient identifier. The imaging test only contains the associated Medicare Benefits Schedule (MBS) codes. Cost information for pathology tests was obtained by matching each test from the record to descriptions in the 2019 MBS Book [1]. We also consulted relevant persons (departmental managers of radiology and pathology, clinical nurse consultant), to ensure valid cost estimations. All tests were costed at 100% of the MBS schedule fees. Costs are in AUD. No adjustments were made for inflation given the source book was current.

Reference

1. Australian Government Department of Health. Medicare Benefits Schedule Book Category 6– Pathology Services. 2019. p. 1-165.
