# Supplementary material for: Associations between pre-surgical daily opioid use and short-term outcomes following knee or hip arthroplasty: a prospective, exploratory cohort study
Source: BMC Musculoskelet Disord. 2020 Jun 22;21:398. doi: 10.1186/s12891-020-03413-z (PMC7310486; doi:10.1186/s12891-020-03413-z)
Supplement: Supplementary file 4 — Additional file 4: Table 3S. Poisson regression for length of stay. [file 12891_2020_3413_MOESM4_ESM.docx]

**ADDITIONAL FILE 4**

**Associations between pre-surgical daily opioid use and short-term outcomes following knee or hip arthroplasty: a prospective, exploratory cohort study**

Justine M Naylor (PhD, BAppSc (Phty)), Natalie Pavlovic (BAppSc (Phty) Hons 1), Melissa Farrugia (BAppSc (Phty)), Shaniya Ogul (BN), Danella Hackett (M Physio, BAppScEXSS), Anthony Wan (MBBS, FANZCA), Sam Adie (BSc(Med) MBBS(Hons) MSpMed MPH PhD FRACS), Bernadette Brady (PhD, MManTher, BAppSc (Phty) Hons 1), Leeanne Gray (Grad Dip Nursing management), Rachael Wright (BAppSc (OccTherapy)), Michelle Nazar (RN), Wei Xuan (MSc MAppStat PhD)

Table 3S. Poisson regression for length of stay

| Parameter | DF | Estimate | Wald 95% Confidence Limits | | Pr > ChiSq |
| --- | --- | --- | --- | --- | --- |
| Daily opioids pre-surgery vs not | 1 | 0.0512 | -0.0590 | 0.1613 | 0.3625 |
| Age, yr | 1 | 0.0100 | 0.0054 | 0.0146 | <.0001 |
| Male vs female | 1 | -0.1464 | -0.2336 | -0.0592 | 0.0010 |
| Total knee vs total hip arthroplasty | 1 | -0.0081 | -0.1022 | 0.0861 | 0.8662 |
| ASA 3 or 4 vs 1 or 2 | 1 | 0.0816 | -0.0037 | 0.1670 | 0.0607 |
| Obese vs non-obese* | 1 | 0.0533 | -0.0341 | 0.1408 | 0.2317 |
| Interpreter required vs not | 1 | 0.1224 | 0.0348 | 0.2099 | 0.0062 |
| Acute complication vs not | 1 | 0.3951 | 0.3032 | 0.4870 | <.0001 |
| Any mental health disorder vs not | 1 | -0.0187 | -0.1283 | 0.0909 | 0.7379 |

Note: The estimate is shown, not the rate ratio; ASA = American Society of Anesthesiologists; * Body mass index < 30 or ≥ 30.
